# Effects of a presumably protective endosymbiont on life‐history characters and their plasticity for its host aphid on three plants

**DOI:** 10.1002/ece3.4754

**Published:** 2018-12-11

**Authors:** Shirong Li, Deguang Liu, Rongfang Zhang, Yingting Zhai, Xianliang Huang, Da Wang, Xiaoqin Shi

**Affiliations:** ^1^ State Key Laboratory of Crop Stress Biology for Arid Areas (Northwest A&F University) Yangling Shaanxi Province China; ^2^ College of Plant Protection Northwest A&F University Yangling Shaanxi Province China

**Keywords:** G‐matrix, insect‐plant interactions, life‐history traits, phenotypic plasticity, plant adaptation, Secondary symbiont

## Abstract

*Hamiltonella defensa* is well known for its protective roles against parasitoids for its aphid hosts, but its functional roles in insect‐plant interactions are less understood. Thus, the impact of *H. defensa *infections on life‐history characters and the underlying genetic variation for the grain aphid, *Sitobion avenae* (Fabricius), was explored on three plants (i.e., wheat, oat, and rye). Compared to cured lines, *H. defensa* infected lines of *S. avenae *had lower fecundity on wheat and oat, but not on rye, suggesting an infection cost for the aphid on susceptible host plants. However, when tested on rye, the infected lines showed a shorter developmental time for the nymphal stage than corresponding cured lines, showing some benefit for *S. avenae* carrying the endosymbiont on resistant host plants. The infection of *H. defensa* altered genetic variation underlying its host *S. avenea*’s life‐history characters, which was shown by differences in heritabilities and genetic correlations of life‐history characters between *S. avenae* lines infected and cured of the endosymbiont. This was further substantiated by disparity in G‐matrices of their life‐history characters for the two types of aphid lines. The G‐matrices for life‐history characters of aphid lines infected with and cured of *H. defensa* were significantly different from each other on rye, but not on oat, suggesting strong plant‐dependent effects. The developmental durations of infected *S. avenae* lines showed a lower plasticity compared with those of corresponding cured lines, and this could mean higher adaptability for the infected lines.Overall, our results showed novel functional roles of a common secondary endosymbiont (i.e., *H. defensa*) in plant‐insect interactions, and its infections could have significant consequences for the evolutionary ecology of its host insect populations in nature.

## 1 INTRODUCTION

All insects are colonized by microorganisms on the exoskeleton and in the body (e.g., gut and hemocoel), and microbiota accounts for up to 1%–10% of insects’ biomass (Douglas, 2015). The majority of symbionts have formed a facultative relationship with their insect hosts, although some are required for their hosts’ survival and reproduction (i.e., obligate mutualism; Donald et al., 2016; Dykstra et al., 2014). For example, only one obligate endosymbiont (i.e., *Buchnera aphidicola*) occurs in most aphid species, including the pea aphid, *Acyrthosiphon pisum* (Harris), but at least eight facultative endosymbionts are well known in *A. pisum* (Douglas, 1998; Oliver, Degnan, Burke, & Moran, 2010; Russell et al., 2013). It's believed that facultative endosymbionts can enhance some biological and ecological traits for their host aphids, such as the frequency of sexual reproduction (Leonardo & Mondor, 2006; Simon et al., 2011), body coloration (Tsuchida et al., 2010), resistance to parasitoid wasps and pathogenic fungi (Oliver, Russell, Moran, & Hunter, 2003; Scarborough, Ferrari, & Godfray, 2005), and the capacity to withstand heat shock (Montllor, Maxmen, & Purcell, 2002; Russell & Moran, 2015). One of the most studied facultative symbionts in aphids is *Hamiltonella defensa* (Oliver et al., 2010; Vorburger, Gehrer, & Rodriguez, 2010; Wang et al., 2016). Some studies have shown that *H. defensa* can protect its host aphids (e.g., *A. pisum* and *Aphis fabae*) against parasitoids to certain extents (Martinez, Weldon, & Oliver, 2014; Oliver et al., 2003; Schmid, Sieber, Zimmermann, & Vorburger, 2012; Vorburger et al., 2010), and the significance of its protective roles is dependent on specific *H. defensa* isolates and associated bacteriophage variants of APSE (*Acyrthosiphon pisum* secondary endosymbiont; Degnan & Moran, 2008a; Oliver, Degnan, Hunter, & Moran, 2009). However, there are also studies showing little or no protective effects of *H. defensa* against parasitoids, and its negative impacts on hosts’ longevity and fecundity (Cayetano, Rothacher, Simon, & Vorburger, 2015; Łukasik, Dawid, Ferrari, & Godfray, 2013), indicating this symbiont can have additional functional roles.

Indeed, research on the cowpea aphid (*Aphis craccivora*) shows that *H. defensa* can be found only in aphid individuals collected from alfalfa, which indicates its infection may be in the benefit of the host aphid in dealing with variable selection pressures on host plants (Brady & White, 2013). Additional evidence of *H. defensa*'s potential roles in insect‐plant interactions includes: (a) clones of *A. pisum* harboring *H. defensa* are much better suited to survive on alfalfa (*Medicago sativa*), while those carrying *R. insecticola* perform better on clover (*Trifolium repens*; Leonardo & Muiru, 2003); (b) the infection of *H. defensa* has been found to be associated with populations of *Bemisia tabaci* (Gennadius) from the MEAM 1 (Middle East‐ Asia Minor 1) group only (Blawid et al., 2015); and (c) most individuals of aphids collected from plants of *Genista* sp. harbor a combination of *H. defensa *and* Serratia symbiotica* (Peccoud, Maheo, Huerta, Laurence, & Simon, 2015). Furthermore, feeding by *H. defensa*‐infected whiteflies (*B. tabaci*) suppressed jasmonic acid‐related defense gene expression, and reduced defense‐related enzyme activities in tomato compared to uninfected ones, indicating that interactions between this symbiont and its hosts could result in the manipulation of induced defenses of plants (Su et al., 2015). However, some research presented no evidence that *H. defensa* had a major direct role in facilitating the pea aphid's utilization of host plants (e.g., *Lathyrus* sp. and *Vicia faba*; McLean, Asch, Ferrari, & Godfray, 2011). Therefore, *H. defensa*'s roles in mediating insect‐plant interactions are still controversial, given the established evidence of its protective roles against parasitoids.

The English grain aphid (*Sitobion avenae *[Fabricius]), a well‐known cereal pest of economic importance all over the world (Gao, Liu, Chen, & Meng, 2014), provides a good model to address the issue. This aphid feeds on a wide range of plants from cereals to wild grasses in the Poaceae, and the frequency of occurrences for the facultative endosymbiont *H. defensa* in *S. avenae* appears to be different on various plants (Gao, 2014; Wang et al., 2016). Thus, in order to determine if the presumably protective *H. defensa* can play significant roles in aphid‐plant interactions, *S. avenae* clones were sampled from two provinces of China, and natural infections of *H. defensa* were detected and manipulated. Specifically, the objectives of this study are to: (a) determine if the infection of *H. defensa* can protect *S. avenae* from parasitoids; (b) explore if the infection of *H. defensa* can have significant impacts on its host's plant use and adaptation.

## 2 MATERIALS AND METHODS

### 2.1 Aphid collection and colony establishment

All aphid colonies were derived from single individuals of *S. avenae*, collected on wheat (*Triticum aestivum* L.) from Qinghai and Shaanxi provinces of China from April to July in 2013 (Supporting information Table S1). The colonies were kept on wheat (*T. aestivum* cv. “Aikang 58”) seedlings in the laboratory as described previously (Dai, Gao, & Liu, 2014). Four microsatellite loci (i.e., Sm10, Sm12, Sm17, and S4aΣ) were used to determine the genotype of collected *S. avenae* clones as detailed in Huang, Liu, Wang, Shi, and Simon (2015) (Supporting information Table S1, for more details also see Simon et al., 1999). The seven clones of *S. avenae* used in the current study were confirmed to be different multi‐locus genotypes using this approach. In order to minimize or eliminate confounding effects from past experience of different host plants, test aphid clones were maintained on “Aikang 58” or at least three generations prior to their life‐history bioassays (Gao et al., 2014).

### 2.2 *Hamiltonella defensa*'s detection

Detection of *H. defensa* was conducted as described previously in Wang et al. (2016). Briefly, the amplification of bacterial 16S rDNAs was conducted using the universal primers 16SA1 (5′‐AGAGTTTGATCMTGGCTCAG‐3′) and 16SB1 (5′‐TACGGYTACCTTGTTACGACTT‐3′). PCR amplicons were then cloned. Sequencing of 16S rDNA fragments was done using the sequencing facility at Sangon Biotech (Shanghai, China). After blast searches, some resulting sequences from certain clones of *S. avenae* were found to be 99% identical with the Genbank sequence of *H. defensa* (e.g., AY907546.1) from the pea aphid (*A. pisum*). We also identified *B. aphidicola*, *R. insecticola*, and *Ricketssia* sp. in this process (data not shown). We compared the resulting *H. defensa* sequences and previously published sequences in GenBank in order to develop diagnostic primers (forward: 5′‐GCGATAAATGCGAATACCAT‐3′; reverse: 5′‐ TTCCCTCGCAGGTTCGCATCC‐3′). These primers were then used in diagnostic PCRs: 94°C for 5 min, and then 35 cycles consisting of 94°C for 0.5 min, 55°C for 1 min, and 72°C for 1.5 min. We found seven *S. avenae* clonal lines which harbored only the target facultative endosymbiont (i.e., *H. defensa*). Antibiotic treatments were applied on these lines to eliminate *H. defensa* (see below) and develop corresponding uninfected lines of the same genetic makeup. Regular diagnostic PCRs and sequencing were conducted to confirm the infection status of *H. defensa* in each test line. The occurrence or loss of *H. defensa* was also assessed on siblings of the aphid individuals used in bioassays of parasitism and aphid life‐history. All of our test aphid clones contained one *H. defensa *strain only (Genbank accession number of the 16S rDNA sequence, KY082763). We used specific primers to determine the presence and type of the bacteriophage APSE (*Acyrthosiphon pisum* secondary endosymbiont) in our test aphid clones according to the studies of Degnan and Moran (2008b), and Weldon, Strand, and Oliver (2013). All our test aphid clones were found to contain the gene fragment *cdtB* (encoding for the cytolethal distending toxin) using diagnostic primers (forward: 5′‐ATATTTTTTTTACCGCCCCG‐3′; reverse: 5′‐CCAGCTTCATTTCTACCACCTC‐3′), suggesting that they all harbored the same phage APSE‐2 (Weldon et al., 2013).

### 2.3 Eradication of *H. defensa* in infected aphid lines

By using the oral administration of antibiotics as detailed in Wang et al. (2016), we cured the seven abovementioned aphid lines which were naturally infected with *H. defensa*. Briefly, we placed cut wheat stems into 1.5 ml Eppendorf tubes, containing 100 μg/ml ampicillin, 50 μg/ml cefotaxime, and 50 μg/ml gentomicin, and then introduced second instar nymphs of *S. avenae* on the wheat stems for feeding about 4–6 days at 20°C (Douglas & Prosser, 1992). Survived aphid nymphs were then reared on wheat seedlings (one‐two leaf) separately. The progeny of these aphid nymphs were checked for the presence or absence of *H. defensa *using the above‐mentioned diagnostic primers.

Clonal lines were then established using the offspring of each test clone whose infections of *H. defensa* had been eliminated. To eliminate the residual effects of antibiotic treatment, cured lines were not allowed for any experiment, and tested for the lack of *H. defensa* for at least six generations (McLean et al., 2011). The seven cured lines and corresponding *H. defensa*‐infected lines were cultured on wheat seedlings (cv. Aikang 58), and regularly tested for *H. defensa* infection status. We also reconfirmed the infection status of *H. defensa *in test aphid lines following parasitism and life‐history bioassays.

### 2.4 Parasitism and life‐history bioassays for infected and cured aphid lines

Bioassays on parasitism of the endoparasitoid, *Aphidius gifuensis *(Ashmead), were performed following the method of Ahmed, Liu, and Simon (2017). Briefly, in order to ensure the occurrence of mating, newly emerged parasitoid pairs (1 female and 1 male) were introduced into a gelatin capsule with 10% honey solution, and kept there for 24 hr. Twenty 3rd instar nymphs of *S. avenae* were transferred onto an experimental wheat seedling (one or two‐leaf plant), which was enclosed with a plastic tube (diameter, 6 cm; height, 15 cm). This developmental stage of *S. avenae* was chosen because it was the favorite stage for *A. gifuensis* in our previous study (Ahmed et al., 2017). A mated pair of the parasitoid (*A. gifuensis*) was then introduced into the plastic cage, and kept there for 8 hr. Test individuals of *S. avenae* were maintained therein for up to 10–15 days (from the day of parasitoid exposure) under the following conditions: temperature, 20 ± 1°C; relative humidity, 65% ± 5%; photoperiod, L16: D8. From day 7, they were examined once daily, and numbers of mummies were tabulated. The experiment was replicated at least six times for each treatment of an aphid clone.

For life‐history bioassays of the seven clonal lines infected and cured of *H. defensa*, we followed the same protocols as described previously (Gao & Liu, 2013; Huang, Liu, Gao, & Chen, 2013). Three plants were tested, and they included wheat (*T. aestivum*, cv. “Aikang 58”), oat (*Avena sativa *L., cv. “Sandle”) and rye (*Lolium perenne *L., cv. “Bison”). Briefly, newly born nymphs (ca. 12 hr) of all experimental lines were introduced onto plant seedlings (one‐two leaf). These seedlings were well covered with a transparent tube (diameter, 6 cm; height, 15 cm) which had a Terylene mesh top for ventilation. Bioassays were conducted in growth chambers with the above‐mentioned conditions. To reduce the potential positional effects, the trays holding test wheat seedlings with inoculated aphids were routinely rotated in the growth chamber. The test new‐born nymphs were monitored daily, and their developmental status, number of offspring produced (offspring removed after counting), and mortality were tabulated until day 10 after the initiation of reproduction for each test individual. We used five to ten replicates for each *S. avenae* line. Test wheat seedlings were watered when needed, and replenished weekly. In terms of fecundity, winged and wingless aphids were shown to have differential performances, so we only used replicates of wingless aphids (the most common morph in our tests) in the following statistical analyses (Łukasik et al., 2013).

### 2.5 Statistical analyses

As detailed previously in Dai et al. (2014) and Wang et al. (2016), three‐way nested analyses of variance (ANOVA) in SAS were utilized to compare developmental durations of 1st to 4th instar nymphs (DT1 to DT4), the total duration of the entire nymphal stage (DT5), and 10 days fecundity. In these analyses, we examined the effects of test plant, treatment (i.e., eradication of *H. defensa*), aphid genotype nested in treatment, and the interactions between the first two factors. Tukey tests were used to separate means at *α* = 0.05 after significant ANOVAs. When needed, data log‐transformations were carried out to meet the requirements (i.e., normality and homoscedasticity) of ANOVAs.

We used clonal genotypes in the bioassays of comparing life‐histories of *S. avenae*. This experimental design allowed us to calculate the total variance of a certain life‐history trait (*V_P_*), which included both within‐genotype components *V_E_* (i.e., environmental variance) and among‐genotype components *V* ***_G_*** (i.e., genetic variance; Dai et al., 2014). Using the restricted maximum likelihood (REML) method, variances (phenotypic or genetic) and covariances of *S. avenae* life‐history traits were assessed in the software VCE (version 6.0.2; Neumaier & Groeneveld, 1998). As described previously in Huang et al. (2015), broad‐sense heritabilities of life‐history characters were evaluated as *H* ^2^ = *V_G_/V_P_*, and genetic correlations between characters *x *and *y* were calculated as *r* = cov (*x*, *y*)/[(*v_x_*) × (*v_y_*)]^0.5^ (cov[*x, y*], genetic covariance of *x *and *y*; *v_x_* and *v_y_*, genetic variance of *x *and *y*, respectively). The structural differences between paired G matrices were determined using the Flury hierarchical method in the software CPCrand as described in Phillips and Arnold (1999). The structural relationship between paired G‐matrices was tested in the following order: unrelated structure, partial common principal components, common principal components, proportionality, and equality. Following Carter, Simon, and Nespolo (2012), the statistical significance of genetic correlations and broad‐sense heritabilities was determined using the likelihood‐ratio tests (LRTs).

As detailed previously in Dai et al. (2014), the amount of plasticity for life‐history characters of different clonal lines on the three test host plants (i.e., wheat, oat, and rye) was calculated as the coefficient of variation using the equation$CV=SD/\overset{¯}{x}\times 100$CV = SD/\bar{x} \times 100CV = SD/\bar{x} \times 100 (*SD*, the standard deviation of each treatment; $\overset{¯}{x}$\bar{x}\bar{x}, the mean of each treatment).

The selective strength of alternative host plants on life‐history character plasticity of infected and cured aphid lines was examined using selection differentials and gradients following Dai et al. (2014). These selection parameters were calculated by using the PROC REG procedure in SAS. Briefly, 10 days fecundity was used as a fitness surrogate for calculating the relative fitness of each test aphid line in different treatments, and all data in this analysis were standardized (i.e., mean zero and unit variance). Simple and multiple regressions were utilized to determine standardized selection differentials (i.e., the total selective strength on a particular trait including both direct and indirect selection) and gradients (i.e., the strength of direct selection only), respectively (for more details, see Lande & Arnold, 1983).

## 3 RESULTS

### 3.1 Differences in parasitism rates and fitness characters

Third instar nymphs of all seven *S. avenae* clones showed no significant differences between treatments (i.e., infected and cured of *H. defensa*) in parasitism rates by the parasitoid (*A. gifuensis*; Supporting information Figure S1).

“Treatment” (i.e., the eradication of *H. defensa*) showed significant effects on DT3, DT5 and 10 days fecundity, although it accounted for relatively little (0.3% to 3.0%) of the total variation of test characters (Table 1). “Test plant” had significant impacts on all test life‐history characters of *S. avenae*, and contributed 2.7% to 30.4% to the total variation. Interactive effects of “treatment” and “test plant” were found for both the total developmental time of nymphs and 10 days fecundity. “Clone” (i.e., different genotypes) nested in “treatment” showed significant effects for all test characters but the developmental time of 1st instar nymphs (DT1), and it explained 4.7% to 25.2% of the total variation.

**Table 1:** Summary of variance components for life‐history characters of *Sitobion avenae* clones showing main effects of treatment, test plant (plant), clone nested in treatment and treatment‐plant interactions

Character	Variance source	*df*	*F*	*p*	% total
DT1	Treatment	1	1.9	0.169	0.6
	Plant	2	4.23	**0.0155**	**2.7**
	Treatment × plant	2	0.46	0.6318	0.3
	Clone (treatment)	12	1.21	0.2763	4.7
	Error	284			91.7
DT2	Treatment	1	3.74	0.0542	1.0
	Plant	2	22.04	**<0.001**	**12.3**
	Treatment × plant	2	2.24	0.1086	1.2
	Clone (treatment)	12	1.86	**0.0389**	**6.2**
	Error	284			79.2
DT3	Treatment	1	7.56	**0.0063**	**2.0**
	Plant	2	22.84	**<0.001**	**11.8**
	Treatment × plant	2	6.75	**0.0014**	**3.5**
	Clone (treatment)	12	3.08	**<0.001**	**9.5**
	Error	284			73.2
DT4	Treatment	1	1.42	0.2349	0.3
	Plant	2	32.46	**<0.001**	**14.3**
	Treatment × plant	2	2.51	0.083	1.1
	Clone	12	8.35	**<0.001**	**22.0**
	Error	284			62.3
DT5	Treatment	1	5.13	**0.0243**	**1.0**
	Plant	2	61.21	**<0.001**	**23.6**
	Treatment × plant	2	5.39	**0.005**	**2.1**
	Clone (treatment)	12	7.95	**<0.001**	**18.4**
	Error	284			54.9
10 days fecundity	Treatment	1	21.97	**<0.001**	**3.0**
	Plant	2	110.25	**<0.001**	**30.4**
	Treatment × plant	2	7.86	**<0.001**	**2.2**
	Clone (treatment)	12	15.23	**<0.001**	**25.2**
	Error	284			39.2

Note

Treatment, with or without antibiotic removal of *Hamiltonella defensa* in aphid clones; significant effects highlighted in boldface type.

**Figure 1:**
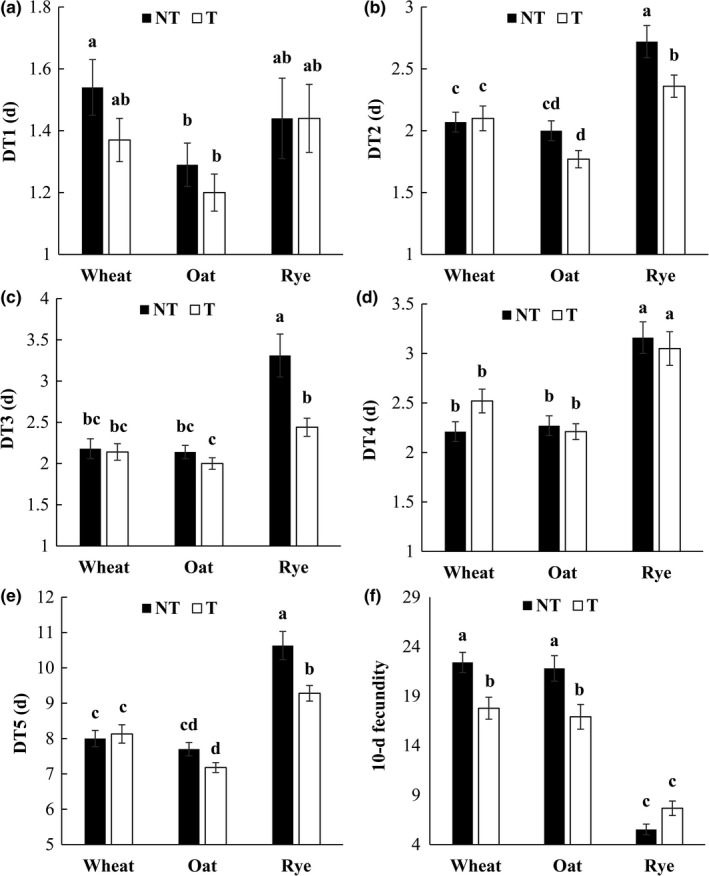
Comparisons of life‐history characters (*SE*) on three host plants for *Sitobion avenae* lines infected and cured of *Hamiltonella defensa *(a, DT1; b, DT2; c, DT3; d, DT4; e, DT5; f, 10‐days fecundity; T indicates aphid lines infected with *H. defensa*; NT indicates corresponding aphid lines with *H. defensa* eradicated; DT1–DT4, the developmental time of 1st to 4th instar nymphs; DT5, the total developmental time of nymphs; different letters above bars of a particular character indicate significant differences among treatments at the *p* < 0.05 level, ANOVA followed by Tukey tests)

No significant differences in DT1 were identified on any test plant between *S. avenae* lines infected with *H. defensa* and corresponding cured lines (Figure 1). The developmental time of 2nd instar nymphs (DT2) of infected *S. avenae* lines was prolonged than that of corresponding cured lines on rye, but not on wheat or oat (*F* = 22.04; *df* = 2, 284; *p* < 0.001). Similarly, infected lines of *S. avenae* showed a longer DT3 (the developmental time of 3rd instar nymphs) on rye compared to corresponding cured lines (*F* = 6.75; *df* = 2, 284; *p* < 0.01). Compared to that on rye, DT3 of infected lines was shortened on oat, but not on wheat. There were no significant differences in DT4 (the developmental time of 4th instar nymphs) between infected *S. avenae* lines and corresponding cured lines on the three test plants. The total developmental time of nymphs for cured *S. avenae* lines was not significantly different from that for corresponding infected lines on wheat or oat, but it was prolonged on rye (*F* = 5.39; *df* = 2, 284; *p* < 0.01).

Cured lines of *S. avenae* showed a higher 10 days fecundity than corresponding infected lines on wheat (*F* = 21.97; *df* = 1, 284; *p* < 0.001). The same pattern was found on oat, whereas there were no significant differences on rye between the two types of *S. avenae *lines. For both cured and infected lines of *S. avenae*, 10 days fecundity on wheat or oat was higher than that on rye (*F* = 7.86; *df* = 2, 284; *p* < 0.001), whereas there were no significant differences in 10 days fecundity between the former two plants for either type of *S. avenae* lines.

### 3.2 Genetically‐based variation

Cured *S. avenae* lines showed significant genetic correlations between DT1 and DT2 (negative), as well as between DT1 and DT4 (positive; Table 2). For these lines, DT3 was positively correlated to DT5, whereas DT4 and DT5 were found to be positively correlated to 10 days fecundity. For *S. avenae* lines naturally infected with *H. defensa*, DT5 was positively correlated to DT1, DT2, DT3, and DT4, whereas 10 days fecundity was negatively correlated with DT2, DT4, and DT5. All the other pairwise correlations between test characters of *S. avenae* were non‐significant.

**Table 2:** Genetic correlations among life history characters for *Sitobion avenae* lines infected (above the diagonal) and cured (below the diagonal) of the endosymbiont *Hamiltonella defensa*

Characters	DT1	DT2	DT3	DT4	DT5	10‐days fecundity
DT1	–	−0.1703	0.1629	0.1875	0.4027^*^	−0.1657
DT2	−0.5204^*^	–	0.1964	0.1892	0.5038^*^	−0.6534^**^
DT3	0.0480	0.1221	–	0.3218	0.6777^**^	−0.3574
DT4	0.5144^*^	0.3761	0.2112	–	0.7675^***^	−0.4331^*^
DT5	0.3264	−0.1324	0.6200^**^	0.3724	–	−0.4668^*^
10 days fecundity	0.3816	−0.1279	0.2548	0.4789^*^	0.4740^*^	–

Note

Genetic correlations were derived from variances calculated from combined data on three test plants (i.e., wheat, oat, and rye).

* *p* < 0.05.

** *p* < 0.01.

*** *p* < 0.001.

Pairwise comparisons were made for G‐matrices of infected *S. avenae* lines and corresponding cured lines using the Flury's method and jump‐up approach (i.e., the hypothesis of unrelated structure was tested at each step in the hierarchy to identify structural differences between the paired G‐matrices; Table 3). The full CPC model best explained the structural differences between G‐matrices of infected and cured *S. avenae* lines when tested on wheat (LRT = 322.3, *p* < 0.001), but the paired G‐matrices were not equal (LRT = 34.2, *p* < 0.05). The G‐matrices for both types of *S. avenae* lines were found to be equal when tested on oat (LRT = 41.1, *p* < 0.001). The structural differences between G‐matrices for *S. avenae* lines infected and cured of *H. defensa *were best explained by the CPC(1) model (meaning only one principal component shared in common) when tested on rye.

**Table 3:** Comparisons of G‐matrices for life‐history characters of *Sitobion avenae* lines infected (T) and cured (NT) of the endosymbiont *Hamiltonella defensa*

G matrices	Test plant	Flury hierarchy		
		LRT	*p*‐value	Verdict
T versus NT	Wheat	35.3	0.026	Full CPC
	Oat	17.1	0.707	Equal
	Rye	51.9	<0.001	CPC(1)

Note

The verdict showed the best model in the Flury hierarchy that explained the structural differences between test matrices; significant deviation from equality for the paired matrices was indicated by *p*‐values; full CPC, all principal components shared in common; equal, no significant differences found between paired matrices; CPC(1), one of the six possible components shared in common.

### 3.3 Fitness character plasticity and selective effects of three test plants

**Figure 2:**
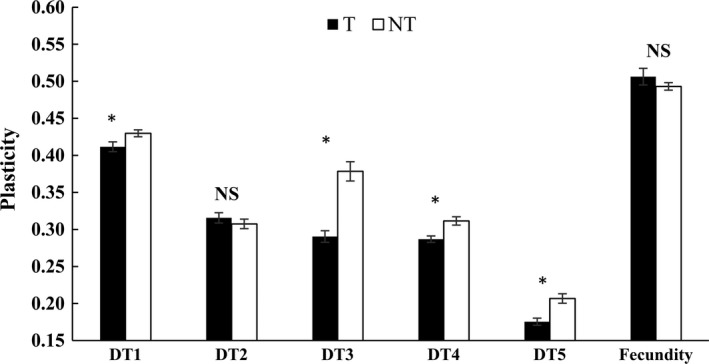
Comparisons of life‐history character plasticities between *Sitobion avenae* lines infected (T) and cured (NT) of the endosymbiont *Hamiltonella defensa* (DT1–DT4, developmental time of 1st to 4th instar nymphs; DT5, total developmental time of nymphs; * and NS, significant and non‐significant differences between T and NT at *α* = 0.05, respectively)

Higher phenotypic plasticity in cured *S. avenae *lines was found for DT1, DT3, DT4, and DT5, compared to corresponding infected lines (Figure 2). No significant differences between the two kinds of *S. avenae* lines were identified in plasticity of DT2 or 10 days fecundity.

The selection of three alternative environments (i.e., wheat, oat and rye) on life‐history character plasticities was analyzed for both infected and cured *S. avenae* lines (Table 4). Under the selection of three alternative plants, lines of *S. avenae* infected by *H. defensa* showed significantly negative differentials for plasticities of all test life‐history characters except DT4. The directional selection gradients of these lines were significant and negative for DT1 and DT2, but not for other characters. Similarly, the corresponding cured lines presented significant and negative differentials for plasticities of all test characters but DT2. All selection gradients for test character plasticities were significant. Among these, selection for plasticities of DT1 and DT5 were negative, whereas it was positive for those of all the other characters.

**Table 4:** Selection coefficients for life‐history character plasticities of *Sitobion avenae* lines infected and cured of *Hamiltonella defensa* on three plants

Character plasticities	Aphid lines infected with *H. defensa*		Aphid lines cured of *H. defensa*	
	Differential	Gradient	Differential	Gradient
DT1	−0.2942***, ***	−0.2664*, *	−0.2164**, **	−0.4191**, **
DT2	−0.4656***, ***	−0.4706***, ***	−0.0742	0.5106***, ***
DT3	−0.2123**, **	0.2022	−0.1869*, *	1.0068***, ***
DT4	−0.1157	0.0456	−0.1936*, *	0.5710***, ***
DT5	−0.4434***, ***	−0.3293	−0.3472***, ***	−2.1308***, ***
10 days fecundity	−0.3246***, ***	0.1028	−0.2544**, **	0.7451***, ***

Notes

DT1 to DT4, developmental time of 1st to 4th instar nymphs; DT5, the total developmental time of nymphs.

* *p* < 0.05.

** *p* < 0.01.

*** *p* < 0.001.

## 4 DISCUSSION

Parasitism rates of *H. defensa* infected lines of *S. avenae* in our study were not significantly different from those of corresponding cured lines (data not shown), despite that *H. defensa* was well known for its protective roles against parasitoids for its host aphid *A. pisum* (Ferrari, Darby, Daniell, Godfray, & Douglas, 2004; Oliver et al., 2009; Oliver, Campos, Moran, & Hunter, 2008). Little protection against parasitoids provided by *H. defensa* could be explained by superparasitism in aphid clones (Donald et al., 2016), but this was unlikely in our experiments. Another explanation for our results can be related to the type of APSE infecting *H. defensa* in aphid clones. All of the test *S. avenae* clones in our study were found to have *H. defensa* infected with APSE‐2. This type of the bacteriophage in pea aphid (*A. pisum*) clones showed partial to little protection against the parasitoid *Aphidius ervi*, whereas those harboring APSE‐3 had complete resistance against the parasitoid (Degnan & Moran, 2008b; Oliver et al., 2009). Our results are consistent with Łukasik et al. (2013) in that the infection of *H. defensa* did not reduce the susceptibility of its host (i.e., *S. avenae*) to parasitoids. However, experienced parasitoid females were found to have ovipositional preference for uninfected individuals of *S. avenae* over infected ones in choice experiments, showing the impacts of *H. defensa* on searching behaviors of the parasitoid involved (Łukasik et al., 2013). Thus, *H. defensa* in our system could still play some protective roles unidentified. In addition, the infection of *H. defensa* had no, partial and complete protection against *A. ervi* parasitism for the pea aphid biotype of *Genista sagittalis*, *Medicago sativa*, and *Genista tinctoria*, respectively (Leclair et al., 2016), showing aphid host biotype‐specific effects. This endosymbiont (i.e., *H. defensa*) was also shown to have essential nutritional roles allowing its host whitefly (*Bemisia tabaci*) to exploit specific legume species (Rao et al., 2015). Such results suggest that this symbiont could play significant roles in insect‐plant interactions.

In our study, the infection of *H. defensa* significantly altered life‐history traits of its host (i.e., *S. avenae*) on the three test plants (i.e., wheat, oat, and rye). It reduced 10‐d fecundity for its host aphid on both wheat and oat, showing physiological costs of carrying the endosymbiont for the aphid on certain plants. Although they had non‐significant changes in fecundity, the infected lines of *S. avenae* on rye showed a significant drop in the host's developmental time (e.g., DT3 and DT5), suggesting some fitness benefits for the host. Our test cultivars of wheat (cv. Aikang 58) and oat (cv. Sandle) were shown to be susceptible to *S. avenae*, whereas rye (cv. Bison) was highly resistant to this aphid (Gao 2014). Similarly, *S. avenae* clones showed much higher fecundity on wheat and oat than on rye in this study. Thus, the infection of *H. defensa* presented a burden (i.e., reduced fecundity, Figure 1f) for *S. avenae* on its susceptible host plants, but a benefit (i.e., shorter developmental time, Figure 1e) on its resistant host plants. Such results suggested that *H. defensa* could have significant impacts in facilitating utilization of certain plants for its aphid host, and this is consistent with the findings of McLean et al. (2011). The differential responses of *H. defensa* infected aphid clones on the three plants also indicated plant‐dependent effects of secondary endosymbionts on the fitness of insect hosts, and this pattern was further substantiated by interactions between “treatment” (i.e., manipulation of *H. defensa* infection status) and “plant” in 10‐days fecundity in the ANOVA. The strong plant‐dependent effects of *H. defensa* infections in this study agree with the findings of Chandler, Wilkinson, and Douglas (2008), Wagner et al. (2015) and Wang et al. (2016). In addition, clear costs to infections with protective bacteria like *H. defensa* have been difficult to identify (Oliver, Smith, & Russell, 2014). In this study, declined fitness of *S. avenae* lines infected with *H. defensa* on wheat and oat indicated a clear cost to the infection of this endosymbiont for this aphid on both plants. Depending on the relative abundance of different host plants, prevalence of *H. defensa* in *S. avenae* populations can vary in different places due to the identified cost of infection. The fitness cost of *H. defensa *infection on *S. avenae*'s preferred plants (i.e., wheat or oat) also suggests that this endosymbiont could have additional functional roles in its host's use of different plants.

Indeed, this endosymbiont significantly influenced other aspects of the life‐history of its host aphid (*S. avenae*) in this study. The genetic correlation between fecundity and the total developmental time of nymphs was significantly positive for cured lines of *S. avenae*, but it was significantly negative for *H. defensa* infected lines. A life‐history trade‐off between developmental time (i.e., DT5) and fecundity was found in infected lines of *S. avenae*, but not in cured lines, indicating that the infection of *H. defensa* could result in genetic variation among *S. avenae* populations. Compared to cured lines, the G‐matrices for life‐history characters of *H. defensa* infected lines were significantly changed on rye and wheat. This provides another line of evidence that genetic variation (and co‐variation) of *S. avenae*'s life‐history characters was changed because of *H. defensa* infections. Such results are consistent with the changing genetic variation of life‐history traits in *R. insecticola* (another common secondary symbiont) infected *A. pisum* and *S. avenae* on different plants (Ferrari, Scarborough, & Godfray, 2007; Wang et al., 2016). One explanation for the phenomenon is that aphid life‐history trade‐offs from the infection of endosymbionts may allow previously less fit genotypes to survive in the population. Additionally, insect hosts can incorporate maternally transmitted endosymbionts as novel functioning genomes, which may contribute to their increased genetic diversity (Feldhaar, 2011). In addition to their changing genetic variation, phenotypic plasticity of test life‐history traits (e.g., DT1, DT3, DT4, and DT5) of *S. avenae* increased in *H. defensa* infected lines. Life‐history trait plasticity was also altered in *R. insecticola* infected lines of *S. avenae* (Wang et al., 2016), suggesting that this phenomenon can be common for secondary endosymbiont‐infected insects. This makes sense because the physiology of plants can be modified as a result of the infection of secondary endosymbionts in insects (Body, Kaiser, Dubreuil, Casas, & Giron, 2013; Feldhaar, 2011), and selective effects of these plants on insects feeding on them can in turn be altered. Direct evidence for this was found in a study where *H. defensa* induced the accumulation of endogenous salicylic acid and concomitant down‐regulation of jasmonic acid‐dependent plant defenses (Su et al., 2015). Therefore, our data indicate that secondary endosymbionts like *H. defensa* can be driving forces for their hosts’ short‐term adaptive evolution on different plants. More studies are needed to assess the effects of *H. defensa* on the stability of G‐matrix of life‐history characters for its aphid host over time and determine its evolutionary implications in the future.

Overall, our results suggest that *H. defensa* could play significant roles in insect‐plant interactions. Although McLean et al. (2011) showed *H. defensa *had no significant effects on the fitness of its host (i.e., *A. pisum*) on different plants, our study agrees with the opposite findings of Brady and White (2013) and Ferrari et al. (2004). One explanation for the inconsistency is that host insects’ phenotypic variation expressed through symbioses should be the outcome of complex interactions among all involved genomes in the presence of various biotic and abiotic stresses in the environment (Leclair et al., 2016). Another mutually nonexclusive explanation is that endosymbionts could play multiple functional roles necessary for enhancing the fitness of their hosts under various environmental threats. Thus, secondary endosymbionts like *H. defensa* may contribute substantially to the acquisition of ecologically important traits for the infected insects, and promote the spread of heritable bacteria in natural insect populations (Duron, 2014). In order to fully appreciate the ecological and evolutionary dynamics of such symbiotic interactions, further work is needed to take into account simultaneously other ecological factors (e.g., high temperature), and variation in symbiont strains.

## CONFLICT OF INTEREST

None declared.

## AUTHOR'S CONTRIBUTIONS

S.L and D.L conceived and designed research. S.L, R.Z, X.H, D.W, and X.S performed research and collected data. D.L, X.H, and S.L analyzed data. D.L and S.L interpreted results and wrote the paper.

## DATA ACCESSIBILITY

All data are included in this manuscript.
