# Supplementary material for: Effects of a presumably protective endosymbiont on life‐history characters and their plasticity for its host aphid on three plants
Source: Ecol Evol. 2018 Dec 11;8(24):13004–13. doi: 10.1002/ece3.4754 (PMC6308870; doi:10.1002/ece3.4754)

Supplementary Figure S1. Parasitism rates of *Aphidius gifuensis* for *Sitobion avenae* clones infected and cured of *Hamiltonella defensa* (third instar nymphs of *S. avenae* were used; T, aphid lines infected with *H. defensa*; NT, corresponding aphid lines with *H. defensa* eradicated; NS, no significant differences between treatments at the $P < 0.05$ level, Student's *t*-tests)

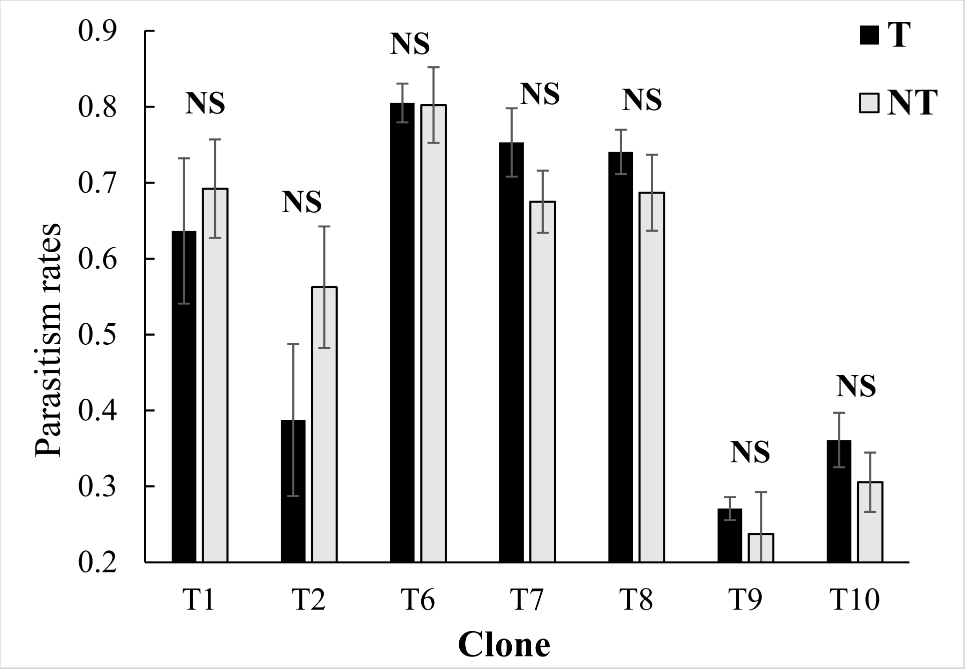

Supplement: Supplementary file 1 [file ECE3-8-13004-s001.pdf]
