# Supplementary material for: Effects of a presumably protective endosymbiont on life‐history characters and their plasticity for its host aphid on three plants
Source: Ecol Evol. 2018 Dec 11;8(24):13004–13. doi: 10.1002/ece3.4754 (PMC6308870; doi:10.1002/ece3.4754)
Supplement: Supplementary file 2 [file ECE3-8-13004-s002.docx]

**Supplementary Table S1. Collection information for seven genotypes^a^ of *Sitobion avenae* infected with *Hamiltonella defensa*** (T, *H. defensa* infected aphid clones; ^a^ aphid genotypes determined by using four microsatellite loci; ^b^ allele sizes at each locus)

| Clones | Collection locality (GPS coordinates) | Microsatellite locus | | | |
| --- | --- | --- | --- | --- | --- |
|  |  | Sm 10 | Sm 17 | Sm 12 | S4aΣ |
| T1 | Fuping Co. in Shaanxi  (E 109° 01′56″, N 34° 46′ 46″) | 157/166^b^ | 96/96 | 149/157 | 154/167 |
| T2 | Chenggu Co. in Shaanxi  (E 107^o^ 16′49″, N 33^o^ 07′ 50″) | 157/163 | 96/100 | 149/155 | 154/167 |
| T6 | Huaying Co. in Shaanxi  (E 110 ^o^ 05′08″; N 34 o 33′ 59″) | 157/166 | 96/100 | 149/157 | 157/163 |
| T7 | Zhouzhi Co. in Shaanxi  (E 108^o^ 13′ 48″, N 34 ^o^ 10′20″) | 152/163 | 100/104 | 151/163 | 158/169 |
| T8 | Huzu Co. in Qinghai  (E 101o 57′ 30″, N 36 o 50′ 37″) | 150/160 | 96/102 | 135/157 | 165/165 |
| T9 | Huzu Co. in Qinghai  (E 102°16′19″, N 36°50′34″) | 155/166 | 96/96 | 147/149 | 161/167 |
| T10 | Huangzhong Co. in Qinghai (101°33′30″, N 36°30′12″) | 157/166 | 96/100 | 149/157 | 165/167 |
