# Supplementary material for: Effects of a presumably protective endosymbiont on life‐history characters and their plasticity for its host aphid on three plants
Source: Ecol Evol. 2018 Dec 11;8(24):13004–13. doi: 10.1002/ece3.4754 (PMC6308870; doi:10.1002/ece3.4754)

Supplementary Table S1. Collection information for seven genotypes[a] of *Sitobion avenae* infected with *Hamiltonella defensa* (T, *H. defensa* infected aphid clones; [a] aphid genotypes determined by using four microsatellite loci; [b] allele sizes at each locus)

Clones	Collection locality (GPS coordinates)	Microsatellite locus			
		Sm 10	Sm 17	Sm 12	S4aΣ
T1	Fuping Co. in Shaanxi (E 109° 01′56″, N 34° 46′ 46″)	157/166[b]	96/96	149/157	154/167
T2	Chenggu Co. in Shaanxi (E 107° 16′49″, N 33° 07′ 50″)	157/163	96/100	149/155	154/167
T6	Huaying Co. in Shaanxi (E 110 ° 05′08″; N 34 o 33′ 59″)	157/166	96/100	149/157	157/163
T7	Zhouzhi Co. in Shaanxi (E 108° 13′ 48″, N 34 ° 10′20″)	152/163	100/104	151/163	158/169
T8	Huzu Co. in Qinghai (E 101o 57′ 30″, N 36 o 50′ 37″)	150/160	96/102	135/157	165/165
T9	Huzu Co. in Qinghai (E 102°16′19″, N 36°50′34″)	155/166	96/96	147/149	161/167
T10	Huangzhong Co. in Qinghai (101°33′30″, N 36°30′12″)	157/166	96/100	149/157	165/167

Supplementary Figure S1. Parasitism rates of *Aphidius gifuensis* for *Sitobion avenae* clones infected and cured of *Hamiltonella defensa* (third instar nymphs of *S. avenae* were used; T, aphid lines infected with *H. defensa*; NT, corresponding aphid lines with *H. defensa* eradicated; NS, no significant differences between treatments at the $P < 0.05$ level, Student's *t*-tests)

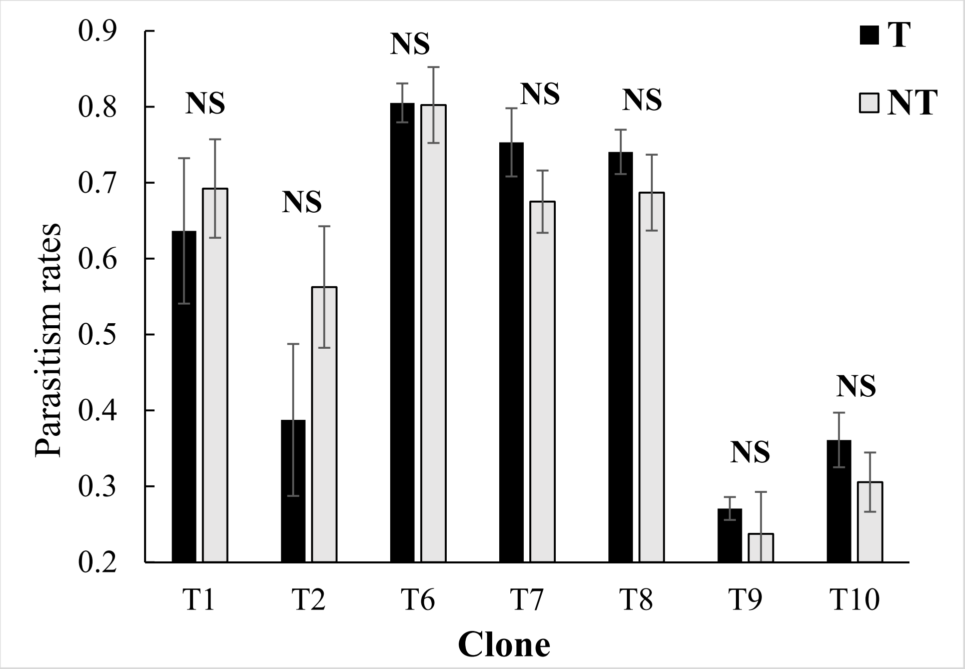

Supplement: Supplementary file 3 [file ECE3-8-13004-s003.pdf]
